# Biomechanical behavior of posterior metal-free cantilever fixed dental prostheses: effect of material and retainer design

**DOI:** 10.1007/s00784-022-04813-2

**Published:** 2022-12-02

**Authors:** Ammar T. Kasem, Abdallah Ahmed Elsherbiny, Manal Abo-Madina, João Paulo M. Tribst, Walid Al-Zordk

**Affiliations:** 1 Fixed Prosthodontics Department, Faculty of Dentistry, Mansoura University, Mansoura, Egypt; 2 Production Engineering and Mechanical Design Department, Faculty of Engineering, Mansoura University, Mansoura, Egypt; 3 Fixed Prosthodontics Department, Faculty of Dentistry, Delta University for Science and Technology, Mansoura, Egypt; 4 Department of Oral Regenerative Medicine, Academic Centre for Dentistry Amsterdam (ACTA), Universiteit Van Amsterdam and Vrije Universiteit, Amsterdam, Noord-Holland, the Netherlands

**Keywords:** Cantilever, Resin bonded, Inlay retained, Fracture, Finite element analysis

## Abstract

**Objective:**

To study the fracture resistance and stress distribution pattern of translucent zirconia and fiber-reinforced composite cantilever resin-bonded fixed dental prostheses (RPFDPs) with two retainer designs.

**Materials and methods:**

Forty human mandibular molars were divided into two groups according to the retainer design. The restorations included a premolar pontic and 2 retainer designs: (D1) inlay ring retainer and (D2) lingual coverage retainer. Each main group was then divided according to the material used (*n* = 10): zirconia (Z) or fiber-reinforced composite (FRC) (F). Restorations were cemented using dual polymerizing adhesive luting resin. All specimens were thermo-cycled (5–55 °C for 10,000 cycles), then subjected to dynamic loading (50 N, 240,000, and 1.6 Hz) and fracture resistance test. The finite element analysis includes the two models of retainer designs used in the in vitro test. Modified von Mises stress values on enamel, dentin, luting resin, and restorations were examined when the restorations failed.

**Results:**

A significantly higher failure load was recorded for zirconia groups (505.00 ± 61.50 and 548.00 ± 75.63 N for D1Z and D2Z, respectively) than for FRC groups (345.00 ± 42.33 and 375.10 ± 53.62 N for D1F and D2F, respectively) (*P* = 0.001). With regard to failure mode, D2 showed a more favorable failure pattern than D1. Model D2 resulted in lower stresses in tooth structure than model D1, and zirconia transmitted more stresses to the tooth structure than FRC.

**Conclusions:**

The lingual coverage retainer (D2) enhanced the biomechanical performance of the restoration/tooth complex. Considering the failure mode and tooth stress, FRC is a promising treatment option when constructing a cantilever RPFDP.

**Clinical relevance:**

Dentists should be aware of the biomechanical behavior during the selection of the material and for the replacement of a single missing mandibular premolar tooth with minimally invasive RBFDP.

## Introduction

Over the last few decades, the possible treatments for replacing missed teeth have increased. The edentulous area can be replaced with traditional porcelain fused to metal, all-ceramic FDPs, and implant-supported restorations [1, 2]. Aside from aesthetic concerns, classic FDPs have the drawback of removing 50–70% of dental tissue during crown preparation, which results in tooth vitality loss in 10% of cases [3–5]. Minimally invasive dentistry has benefited from advancements in dental adhesives with improved bond strength [6]. The RBFDPs, first introduced in 1973, is a minimally invasive solution for replacing a missed tooth [7].

The FDP might be a 3-unit (fixed–fixed) or 2-unit (cantilever) [8] that relies on the periodontal condition of the abutment teeth, the position of the missed tooth, occlusion of the patient, and para-functional habits. Cantilever RBFDPs provide several advantages, including the conservation of tooth structure, easier maintenance of oral hygiene, and low cost [9]. It was previously considered that with a single retainer, the pontic always displaces with its abutment tooth, preventing shear and torque loads that could eventually lead to debonding [10, 11].

The prosthesis material, abutment preparation design, and adhesive material employed all have a role in the clinical success of minimally invasive FDPs [12, 13]. Longer clinical longevity can be expected when using rest seats to prevent apical displacement and proximal boxes to limit the path of insertion and enhance the adhesive surface [14]. However, these adjustments require additional tooth reduction in order to provide retention and resistance forms [15, 16]. The preparation of RBFDPs includes a number of clinical and laboratory procedures that, if not carried out correctly, can have an impact on the treatment’s outcome and durability [17].

Several materials are available to be used as restorations, which may raise questions on which one would have the optimum biomechanical performance, provided that RBFDPs have less adhesive area than a whole crown [18]. According to the literature, posterior RBFDPs have a higher risk of mechanical failures than anterior prostheses [19]. Several materials can be used with posterior RBFDPS, and each material might react mechanically differently to the same applied masticatory force [17, 20]. Early reports of RBFDPs made of metal-ceramic demonstrated promising results, but complications with debonding and caries formation were reported to be the most common observations.

Since the aesthetic aspect of dentistry gained more attention, metal-free new materials such as lithium disilicate and zirconia are also suggested by the manufacturers for the indication of RBFDPs [4, 21]. In vitro studies, comparing the fracture resistance of metal-ceramic and zirconia RBFDPs exhibited higher values for the latter [22]. A recent clinical study [23] evaluated the outcome and survival rate of RBFDPs made of 3Y-TZP ceramic with a cantilever design in the replacement of missing canines, premolars, and molars. The results were promising due to excellent clinical outcomes resulting from a survival rate of 100% and a success rate of 96.3% over a mean observation time of 53 ± 39 months. In a systemic review [24], it was reported that cantilever all-ceramic RBFDPs had a higher survival rate, lower debonding rate, and fracture rate compared with two-retainer all-ceramic RBFDPs. The clinical performance of all ceramic Y-TZP RBFDPs replacing maxillary and mandibular teeth in the anterior and posterior areas demonstrated an 82.7% survival rate over 3 years [21] and 70% of clinical retention after 10 years [6]. Also, the clinical performance of all-ceramic cantilever RBFDPs was shown to be better than that of those fixed–fixed after 5 years [25].

Although ceramics have various advantages, they have lower tensile and bending strength than metals. When the strength of the ceramic material is surpassed, sudden breakage or cracking occurs due to its brittleness. In recent years, the indications for using polymers in dentistry as an alternative to ceramics have grown [25]. FRC materials have gained popularity because of their high fracture toughness associated with tooth-colored matching and the need for minimally invasive preparation [26]. Moreover, due to the anisotropic nature of FRCs, such RBFDPs could be considered more biomimetic options as opposed to other therapy options [25]. On the other hand, the existing FRC RBFDP designs do not have a long lifespan. Delamination, debonding, and fracture have been described as the most common clinical failure modes of FRC RBFDPs [27]. The key criteria determining the performance of FRC are fiber volume, fiber position, and fiber orientation, which have been reported through numerous load-to-failure testing [27, 28].

Most FRC dental materials contain three distinct constituent parts: the continuous phase (which is also named matrix), the dispersed phase (formed by the fibers, generally glass fibers), and the interphase area [29]. TriLor (Bioloren, Saronno, Italy) is a high-performance biocompatible 3D thermosetting FRC composed of epoxy resin matrix (25% vol) and multi-directional glass fiber reinforcement (75% vol). Because of its flexing and bending capacity under stress, TriLor represents an ideal milled composite for implant-supported restorations and creates resilient frames/substructures for zirconia, lithium disilicate, acrylics, and composites due to its characteristics that resemble natural. This material presents adequate tensile strength (380 MPa), flexural strength (540 MPa), modulus of elasticity (26 GPa), and compression strength (perpendicular) (530 MPa) to be used as prosthetic material [29, 30].

To improve the findings of in vitro tests, finite-element analysis (FEA) can be used to complement these results [31]. FEA provides a simulation of the behavior of various materials, methodologies, and designs numerically in terms of displacement and stress distribution with various loading conditions. It allows to assess and measure the biomechanical features of restorative materials, as well as the supporting oral structures [32, 33]. Till today, little information is available on the outcomes of posterior metal-free RBFDPs. Furthermore, data on FRC RBFDPs generally are lacking in the literature. This study was carried out to evaluate the load-bearing capacity and stress distribution pattern of two designs of minimally invasive RBFDP replacing missing mandibular second premolar cantilever on mandibular molar using zirconia or FRC by means of in vitro analysis and 3D FEA. The proposed first null hypothesis was that the design has no effect on the pattern of stress distribution and failure load of cantilever RBFDPs in the posterior region. The second null hypothesis was that the material has no impact on the pattern of stress distribution and failure load of cantilever RBFDP in the posterior region.

## Materials and methods

The protocol of this study was accepted by the Ethical Committee, Faculty of Dentistry, Mansoura University (code: M02080921/2021. The sample size was based on the statistical power of 90% performed with a 95% confidence interval using a statistical tool (OpenEpi, version 3, open-source calculator-SSMean). The subsequent pilot study data (*n* = 10) were inserted in the tool for calculation: confidence interval: 95%, power of 90%, the mean and standard deviation of the group that presented the higher mean (548.00 ± 75.63 N), the mean and standard deviation of the group that presented the lower mean (345.00 ± 42.33 N). The total number of samples to show statistical differences between both mean values was defined as 4. However, the present investigation considered 10 specimens per group, therefore considering all the specimens evaluated during the pilot study.

### In vitro study

Forty human-sound mandibular molars extracted for orthodontic or periodontal reasons with a similar dimension and shape were collected, and the patients signed informed consent to allow using their extracted teeth. Teeth that were cracked or carious were excluded [34]. The teeth inspection has been carried out through visual inspection and during the preparation. With the aid of the transillumination technique, a fiberoptic transilluminator was applied directly to the tooth surface. All other light sources were eliminated, and the light beam was positioned perpendicular to the plane of the suspected crack. If there is a crack, the light would be blocked and it would be visible.

Finally, the selected teeth were disinfected with 1:10 diluted sodium hypochlorite (5.25%) [35]. To avoid dehydration, the teeth were kept in distilled water at room temperature during the testing period. The teeth were embedded vertically within an acrylic resin block to assist handling during preparation, scanning, and cementation procedures. Using the transitional wax technique, a consistent layer (0.3 mm) simulating the periodontal ligament was applied around the roots with light-body impression material (Ghenesyl Superlight, Lascod, Italy) and then encased by an acrylic resin cylinder block (26 mm diameter and 20 mm height) [16, 32].

**Fig. 1:**
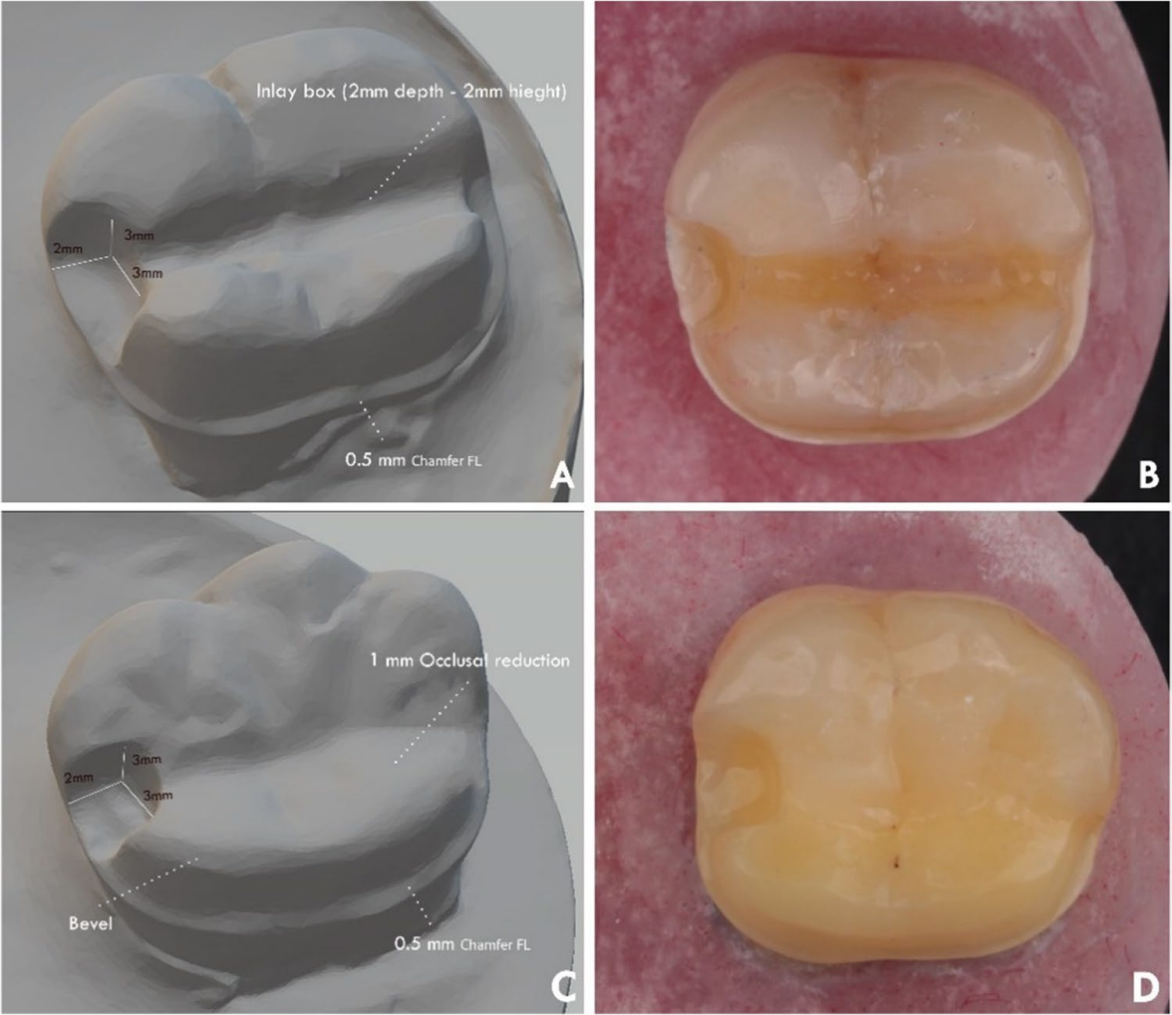
Illustrations showing the preparation designs. **A** and **B** D1: Inlay ring retainer and **C** and **D** D2: lingual coverage retainer

After mounting, the teeth were randomly distributed into four groups of 10 specimens each. According to the preparation designs, the teeth were allocated into two main groups randomly (*n* = 20): design 1 (inlay ring retainer) and design 2 (lingual coverage retainer). Each main group was then divided into 2 subgroups (*n* = 10) according to the material used: Z (3Y-TZP zirconia) and F (FRC). The description of various preparation designs and materials of this study is shown in Table 1. Generally, the two preparation designs (Fig. 1) have a mesial box and lingual chamfer finish line. A specially designed diamond stone (845KR 025, Oekodent, Germany) was used to standardize the proximal box preparation for all teeth. Depth grooves were marked first using a depth cutter (0.5 mm), and the teeth were prepared with 1 mm diameter tapered stone with a round end (#850–016 Long Round End Taper FG × 5, Komet, Germany) mounted in dental surveyor with a convergence angle of 6°. The inlay box of D1 was prepared with a dimension of 2 mm in diameter and 2 mm in depth, with a lingual chamfer finish line (0.5 mm) connected with the inlay box in the central groove. In D2, the preparation included lingual cusp coverage with 1 mm cuspal reduction and 0.5 mm chamfer finish line. All preparation was adjusted to be in enamel [36] except in proximal and inlay boxes, which were adjusted in dentin.

**Table 1:** Description of the different preparation designs investigated in this study

Preparation design	Description	Material	Subgroup	*N*
D1 Inlay ring retainer	Basic preparation design with a mesial box (3 mm height, 3 mm width, and 2 mm depth) Lingual chamfer finish line (0.5 mm) connected with inlay box (2 mm height and depth) in the central groove The lingual cusps are not covered	Z; Monolithic high translucent Zirconia (KATANA HT, Kuarary Noritake, Japan) F; Fiber-reinforced composite (TriLor, Bioloren Srl, Italy)	D1Z	10
			D1F	10
D2 Lingual coverage retainer	Basic preparation design with a mesial box (3 mm height, 3 mm width, and 2 mm depth) Lingual cusps coverage with 1 mm cuspal reduction and 0.5 mm chamfer finish line The lingual half of the abutment is covered		D2Z	10
			D2F	10

For standardization of preparation, the depth grooves were marked first, and the teeth were prepared using a dental surveyor with a convergence angle of 6°. The preparation dimensions were then checked by a putty index, which was taken prior to the preparation [37]. CAD/CAM (computer-aided design/computer-aided manufacturing) software was then used to check the dimensions of preparation and to standardize all restoration thicknesses and diameters. All specimens were scanned using a 3D optical scanner (Identica hybrid, Medit Dent., Korea) with triple camera scanning and a blue LED light source. Full-contour restorations were designed using compatible software, exoCad DentalCAD, version 2.2–6656/64 (exoCad GmbH, Germany). The marginal fit of the restorations was fixed to exactly 20 μm, and the internal cement gap was set at 60 μm [31]. The connector area to the cantilever pontic was adjusted for all designs at 3x3 mm [9]. The pontic dimensions were adjusted for all specimens on the software (7 mm buccolingually, 6 mm mesiodistally, 6 mm occluso-gingivally, and 2 mm space between pontic and acrylic block).

All zirconia (KATANA HT, Kuarary Noritake, Japan) and FRC (TriLor, Bioloren Srl, Italy) restorations were dry milled, and the enlarged zirconia restorations were then sintered at 1530 °C using a special furnace (Vita-Zyrcomat, Vita Zahnfabrik, Germany) [9]. The polishing of FRC was done using silicone rubbers at 8–10,000 rpm and then diamond paste with a brush, according to the manufacturer’s instructions. Before bonding, zirconia and FRC restorations were airborne particles abraded according to the manufacturer’s instructions with 50 μm Al_2_O_3_ powder at 0.25 MPa (distance = 10 mm, duration = 10 s, and angle = 45°) [9, 38].

All restorations were immersed in distilled water and cleaned in an ultrasonic bath for 5 min (CODYSON ultrasonic cleaner CD-4820, Shenzhen Codyson Electrical Co., Ltd, China), then left to dry for about 10 min. After drying with air, the intaglio surface received a layer of MDP-containing primer (CLEARFIL™ Ceramic Primer Plus, Kuraray Noritake Dent., Japan) that was uniformly applied using a micro-brush. The primer was then dried with air for 5 s as per the manufacturer’s recommendation. Before bonding, the teeth were prepared using 37% phosphoric acid (K-etchant, Kuraray Noritake Dent., Japan) to etch the enamel for 30 s and dentin for 15 s [36]. After cleaning with water spray, the teeth were then conditioned with an adhesive system containing MDP (Panavia V5 Tooth Primer, Kuraray Noritake Dent., Japan), and the prepared restorations were bonded with a dual-polymerizing adhesive luting resin (clear Panavia V5, Kuraray Noritake Dent., Japan). An oxygen protection gel (Oxyguard II) was applied around the margins before complete curing for 40 s [9].

All of the cemented specimens were kept in distilled water (37 °C) for 24 h [1], then exposed to 10,000 cycles of thermal cycling (Thermo-cycler TC21, ROBOTA, Egypt) with a 15-s dwell period in each water bath and 5-s transmission time to mimic approximately 1 year clinically [1, 40]. Using a chewing simulation unit (Chewing Simulator CS-4.4, SD Mechatronik, Germany), they were then subjected to 240,000 cycles (frequency = 1.6 Hz) to simulate a uni-directional vertical force of 50 N [32]. The load was applied vertically in the distal fossa of the pontic [27] with a 5-mm ball-shaped antagonist contacting the lingual and buccal cusps. Following the artificial fatigue tests, all specimens were exposed to the test of the fracture using a universal testing machine (UTM) (3365, Instron Ind., USA). The load was applied with a 5 mm stainless steel ball perpendicular to the occlusal surface of the pontic [1] at the mesial fossa, contacting the buccal and lingual cusps [27], with a cross-head speed of 0.5 mm/min [1]. In order to avoid accidental peak stresses, a 0.5 mm tin foil was placed between the ball and restorations during force application [9, 41]. The maximum load of failure was listed in newton (N) using the machine software (Bluehill 3, Instron Industrial, USA). The fractured specimens were assessed qualitatively using a stereomicroscope at a magnification of × 40. Failure modes were described and classified in Table 2 based on a three-examiner agreement. A standard scanning electron microscope (SEM) (JSM 6510 LV, JEOL-Ltd., Japan) was then used to analyze pictures of the fractured surfaces of the specimens, which were sputter coated with a 10 nm layer of the gold–palladium system.

**Table 2:** Classification of the failure modes (CEJ: cemento-enamel junction)

Type	Failure mode	Description
I	Favorable (non-catastrophic/repairable)	• Debonding of the restoration without fracture • Fracture of the restoration without displacement (no loss of adhesion) • Fracture of the restoration with displacement (loss of adhesion) • Fracture of the restoration/tooth complex above the CEJ
II	Unfavorable (catastrophic/non-repairable)	• Fracture of the restoration /tooth complex below the CEJ • Root fracture with only restoration displacement (no restoration fracture), which requires tooth extraction

All data was analyzed in the IBM SPSS statistical software (version 22, IBM Co., USA). After confirming normality with Shapiro–Wilk test, the mean (standard deviation), maximum, and minimum values were used to describe quantitative data. The number and percentage were used to describe the qualitative data. For analytical statistics, the interaction effect was determined using a two-way ANOVA test.

### Finite-element analysis

To complement the results of the in vitro study, a 3D finite-element analysis was established to assess the stress magnitude of each group [31]. This approach was used to analyze the mechanical behavior of structures and the pattern of stress distribution in restorations, luting resin, and tooth structure under the application of axial loading. Two finite-element models representing the two preparation designs similar to those in the in vitro setup were generated.

For the model design, the mesh obtained by the 3D optical scanner was converted into NURBS (non-uniform rational basis spline) using CAD software (3D CAD SOLIDWORKS Premium 2019 SP5.1, Dassault Systèmes, France). In this step, anatomical references such as cusp ridges, cusp tips, marginal ridges, and grooves were used to create polylines on the mesh. A similar approach was used to determine the enamel and dentin tissues. The intersection of three or four polylines was used to create a network surface. For each abutment tooth, the final volumetric model was obtained by joining contacting surfaces without non-formed edges or gaps. The cement layer was uniformly designed with a constant thickness of 60 μm [31]. For the different RBFDPs designs, the virtual STL generated by the Dental CAD (exoCad GmbH, Germany) was exported and converted into volumetric models using a reverse engineering approach based on automated volume generation [31]. Each solid structure was modeled separately and joined by contacting surfaces in the CAD. An acrylic resin cylinder block (26 mm diameter and 20 mm height) for teeth fixation was modeled with a PDL simulation (0.3 mm) around the roots [32].

The finite-element mesh was created and revised after the 3D solid models for the two designs were obtained. Each model was subdivided into four-node tetrahedral or eight-node hexahedral solid elements, each with three degrees of freedom [31, 32]. After performing the convergence test with a 10% discrepancy between the values displayed at a specific position in the mesh, different numbers of elements and nodes were achieved. Model D1 was composed of 77,244 elements and 98,613 nodes, while model D2 has 41,389 elements and 72,677 nodes. Mechanical parameters such as Poisson’s ratio and Young’s modulus of enamel, dentin, luting resin, and restoration materials were determined and reported in accordance with the literature and the material’s manufacturer (Table 3). Young’s modulus is a constant of the capacity of a material to resist changes in length under tensile or compression, whereas Poisson’s ratio is the deformation of a material in directions perpendicular to the direction of loading [43].

**Table 3:** Properties of the materials used in the finite-element models

Structure (tissue/material)	Elastic modulus (MPa)	Poisson’s Ratio (ν)	Tensile strength (MPa)	References
Enamel	84,100	0.33	11.50	[21, 31]
Dentin	18,600	0.31	98.70	[21, 31]
Zirconia	210,000	0.26	445	[23, 30, 42]
Fiber reinforced composite	26,000	0.33	380	[Manufacturer]
Luting resin	12,000	0.33	30	[Manufacturer, 21, 41]
Periodontal ligament	69	0.45	-	[31]
Acrylic resin	2900	0.31	-	[31]

All interfaces were considered perfectly bonded, and the model structures were assumed to be linearly elastic, isotropic, and homogenous for simplification and to reduce the processing time [44]. In the present study, Young’s modulus used for the FRC group is considered an isotropic behavior with constant and proportional deformation in all directions of the material. However, it is important to note that in the real scenario, without simplifications, the FRC behaves as an anisotropic material, showing one value of stiffness for each coordinate axis in the 3D system [45, 46]. Both models were fixed by the fact that the nodes of the resin cylinder (mesial, distal, and bottom surfaces) were equal in all directions, assuming *x* = *y* = *z* = 0 [26]. A compressive static load was vertically and occlusally applied at the mesial fossa, touching the buccal and lingual cusps [27].

According to the parameters used for the in vitro analysis and the resultant failure loads for the tested groups, the load cell of force was adjusted at about 505 N for model D1Z, 345 N for model D1F, 548 N for model D2Z, and 375.10 N for model D2F. A spherical solid, rigid, and material with a diameter of 5 mm was used as an antagonist to apply a compressive load (speed of 0.5 mm/min) until fracture or visible plastic deformation occurred [44, 46]. The modified von Mises (mvM) stress was selected as an analysis criterion since it can be used to evaluate the strength of materials under complex stresses [32]. mvM on tooth structures, restorative materials, and luting resin were examined independently for all analyzed models in megapascals (MPa). If the comparable mvM stresses exceed the material’s tensile strength, the material will fail [43]. The results of the calculations are displayed through colorimetric maps of stress distribution.

## Results

### In vitro results

After artificial aging, all specimens survived with no visible signs of early failure, resulting in a 100% rate of survival for all groups. The mean failure load for group D1Z was 505 ± 61.50 N and 345.00 ± 42.33 N for group D1F. Considering the D2 groups, the mean failure load value of 548.00 ± 75.63 N was recorded for zirconia, while FRC resulted in a mean failure load of 375.10 ± 53.62 N (Table 4). The highest mean failure load was detected among group D2Z. Two way ANOVA test (Table 5) revealed a statistically significant effect of the material, while there is no statistically significant difference regarding the RBFDPs design. Combined effect design and material illustrate a non-statistically significant effect on failure load, and 69.5% of failure load is affected by the change of material only (*R*^2^ = 0.695).

**Table 4:** Descriptive statistics of failure load values (*N*) with distribution of failure modes among tested groups. Failure load data are expressed as mean, standard deviation, minimum, and maximum, while failure mode data are expressed as number and percentage

Group	Failure load				Failure mode			
	Mean	SD	Min	Max	Favorable (I)		Unfavorable (II)	
					*N*	%	*N*	%
D1Z	505.00	61.50	425	592	7	70%	3	30%
D1F	345.00	42.33	283	409	8	80%	2	20%
D2Z	548.00	75.63	409	650	10	100%	0	0%
D2F	375.10	53.62	319	460	10	100%	0	0%

**Table 5:** Two-way ANOVA test to detect the effect of interaction between preparation design and material on the failure load

Dependent variable: failure load/*N*					
Source	Type III Sum of squares	df	Mean square	*F*	*P*-value
Corrected model	290,831.075^a^	3	96,943.692	27.362	0.001
Intercept	7,859,709.025	1	7,859,709.025	2.218E3	0.001
Material	277,056.025	1	277,056.025	78.198	0.001
Preparation design	13,359.025	1	13,359.025	3.771	0.060
Material * preparation design	416.025	1	416.025	0.117	0.734
Error	127,548.900	36	3543.025		
Total	8,278,089.000	40			
Corrected total	418,379.975	39			

a. *R*-squared = 0.695 (adjusted *R*-squared = 0.670)

**Fig. 2:**
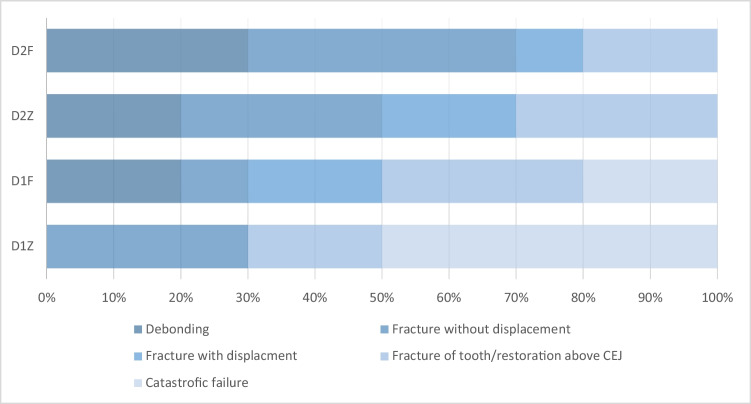
Failure mode analysis of different tested groups

**Fig. 3:**
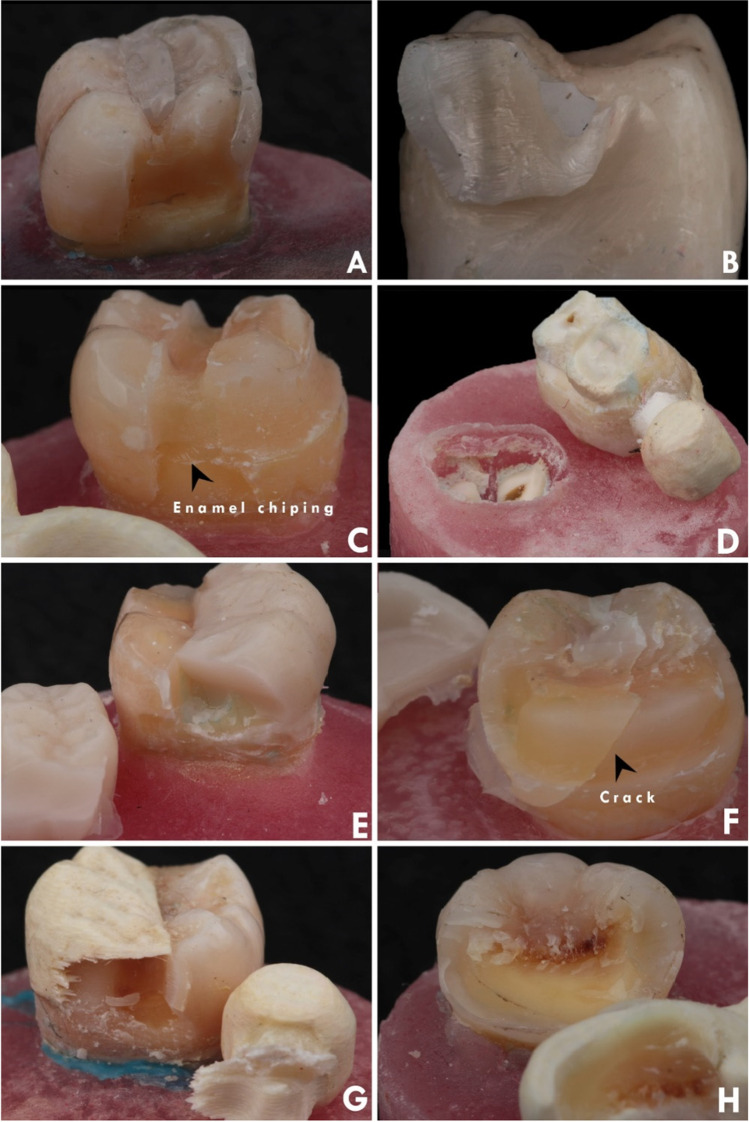
An example image showing the failure mode of specimens. **A** and **B** Fractured zirconia in group D1Z. **C** Debonded restoration with chipped enamel in group D1F. **D** Catastrophic fracture below CEJ in group D1F. **E** Fractured zirconia at connector area in group D2Z. **F** Debonded zirconia with tooth crack in group D2Z. **G** Fractured restoration in group D2F. **H** Debonded restoration with part of the tooth in group D2F

**Fig. 4:**
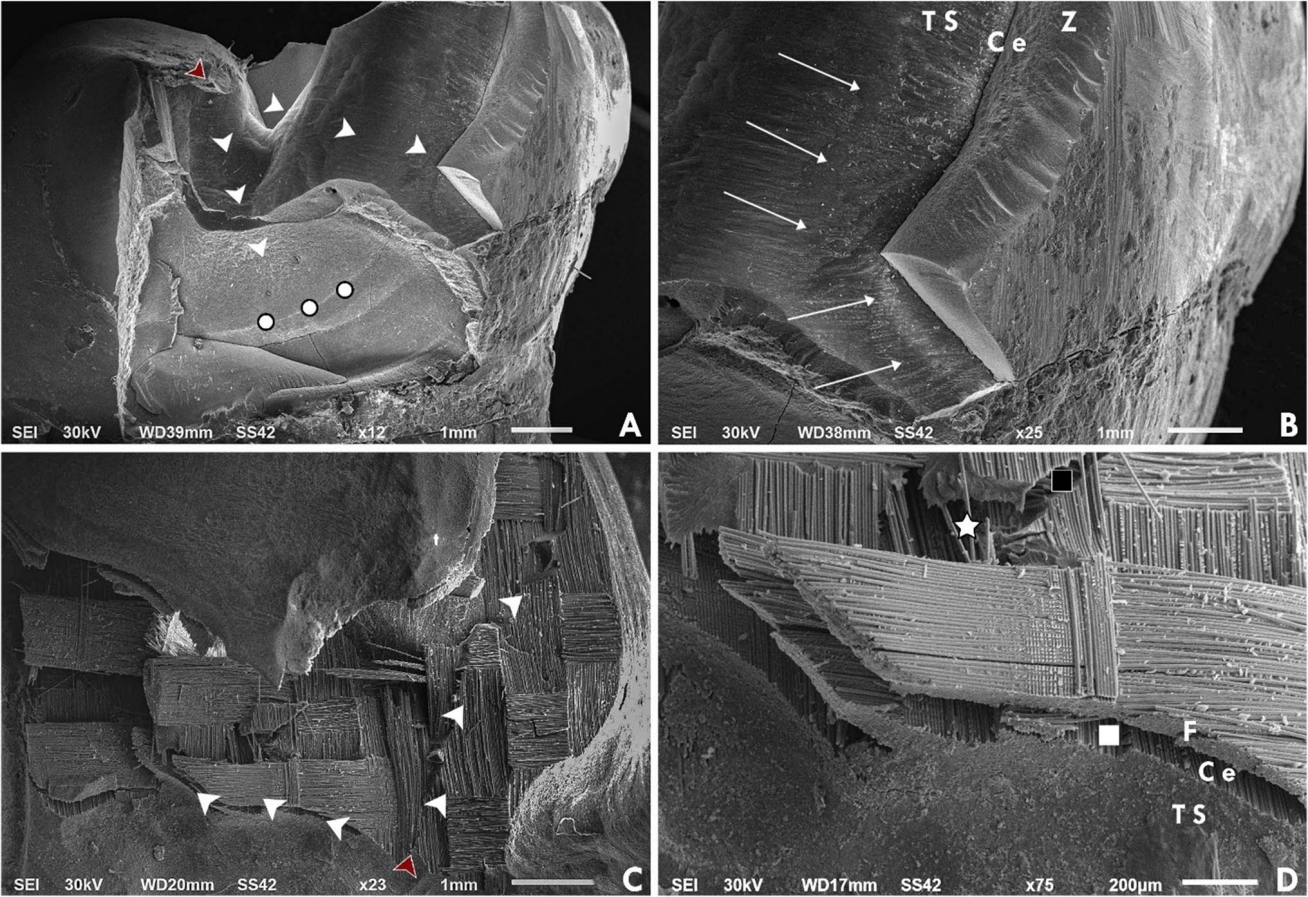
SEM images of failed restorations. **A** and **B** Showing the origin of fracture start from the tooth structure and propagated to zirconia in model D1Z. **C** and **D** showing the origin start from the cement line and cracks propagated through the bundles of the fibers in model D2F. Red arrowhead, failure origin; white arrowhead, direction of crack propagation; arrows, hackle lines; circle, arrest lines; star, fiber pulled out; black square, cohesive failure; white square, adhesive failure; TS, tooth structure; Ce, cement line; Z, zirconia; F, FRC

Failure mode analysis for all tested groups is shown in Table 4 (Fig. 2). Stereomicroscopic analysis (Fig. 3) revealed that 50% of group D1Z and only 20% of group D1F showed catastrophic failure, including tooth fracture below the CEJ. Although several fracture patterns were identified, the majority of specimens were fractured at the thin region of the restorations. Group D1F showed a high rate of debonding (loss of adhesion without fracture). Regarding group D2, all restorations showed only favorable fracture patterns. Most of the restorations of group D2Z were fractured at the connector area, while in group D2F, most of the restorations were fractured without displacement. SEM analysis (Fig. 4) showed that the origin of fracture was mostly located at the occlusal surface, mostly in the connector region for zirconia subgroups, then propagated in the apical direction. The direction of crack propagation was determined with the presence of hackle lines. For FRC subgroups, most fractured specimens showed the main origin of the failure was located at the tooth-restoration interface and propagated in the coronal direction. Cohesive and adhesive failure of the fibers can be seen with fibers pulled out.

## Distribution of stresses in FEA

The maximum mvM of enamel, dentin, cement, and restorative materials for studied models are summarized in (Table 6).

**Table 6:** Maximum modified von Mises stress (mvM) values (MPa) for enamel, dentin, and luting resin for all studied models

Structure (Tissue/material)	Maximum modified von Mises stresses (mvM)			
	D1Z	D1F	D2Z	D2F
Enamel	11.23	11.00	4.00	0.98
Dentin	15.80	11.00	6.40	1.07
Zirconia	27.35	-	32.70	-
Fiber reinforced composite	-	18.00	-	20.70
Luting resin	9.84	20.66	24.09	29.01

### Distribution of stresses in enamel (Fig. 5)

**Fig. 5:**
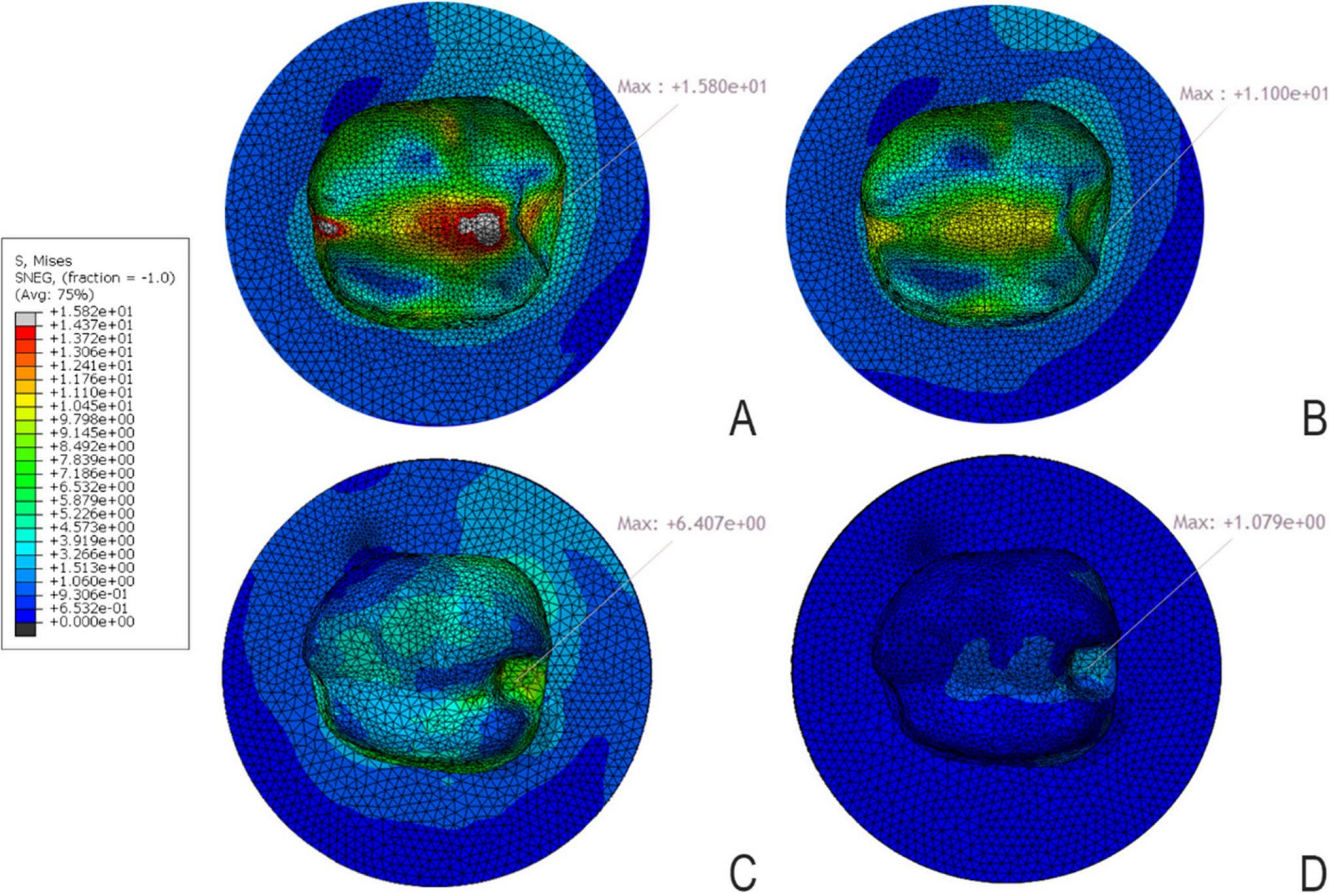
mvM stress distribution pattern for tooth structure for **A** model D1Z, **B** model D1F, **C** model D2Z, and **D** model D2F. Arrows refer to high stresses in CEJ

Model D1 had a higher maximum stress value (11.23 MPa for D1Z and 11.00 for D1F) than model D2 (4.00 MPa for D2Z and 0.98 MPa for D2F). All these values were lower than the enamel tensile strength (11.50 MPa). It means that model D1 transmitted stresses to enamel 4 times more than model D2 in the case of zirconia and 11 times in the case of FRC. The pattern of stress distribution showed more eventual stress distribution for FRC than for zirconia subgroups.

### Distribution of stresses in dentin (Fig. 5)

For mvM analysis, D1 models showed higher maximum stress values (15.80 MPa for D1Z and 11.00 MPa for D1F) than D2 models (6.40 MPa for D2Z and 1.07 MPa for D2F). This means that the stress value of model D1Z was greater about 3 times than that of D2Z and for D1F was about 10 times greater than that of D2F. Also, this means that zirconia transmitted more stresses to the tooth structure than FRC. The mvM stress values for all models did not reach the tensile strength of the dentin (98.70 MPa). In terms of stress distribution, D1 models revealed the concentration of maximal stresses at CEJ.

### Distribution of stresses in the restoration (Fig. 6)

**Fig. 6:**
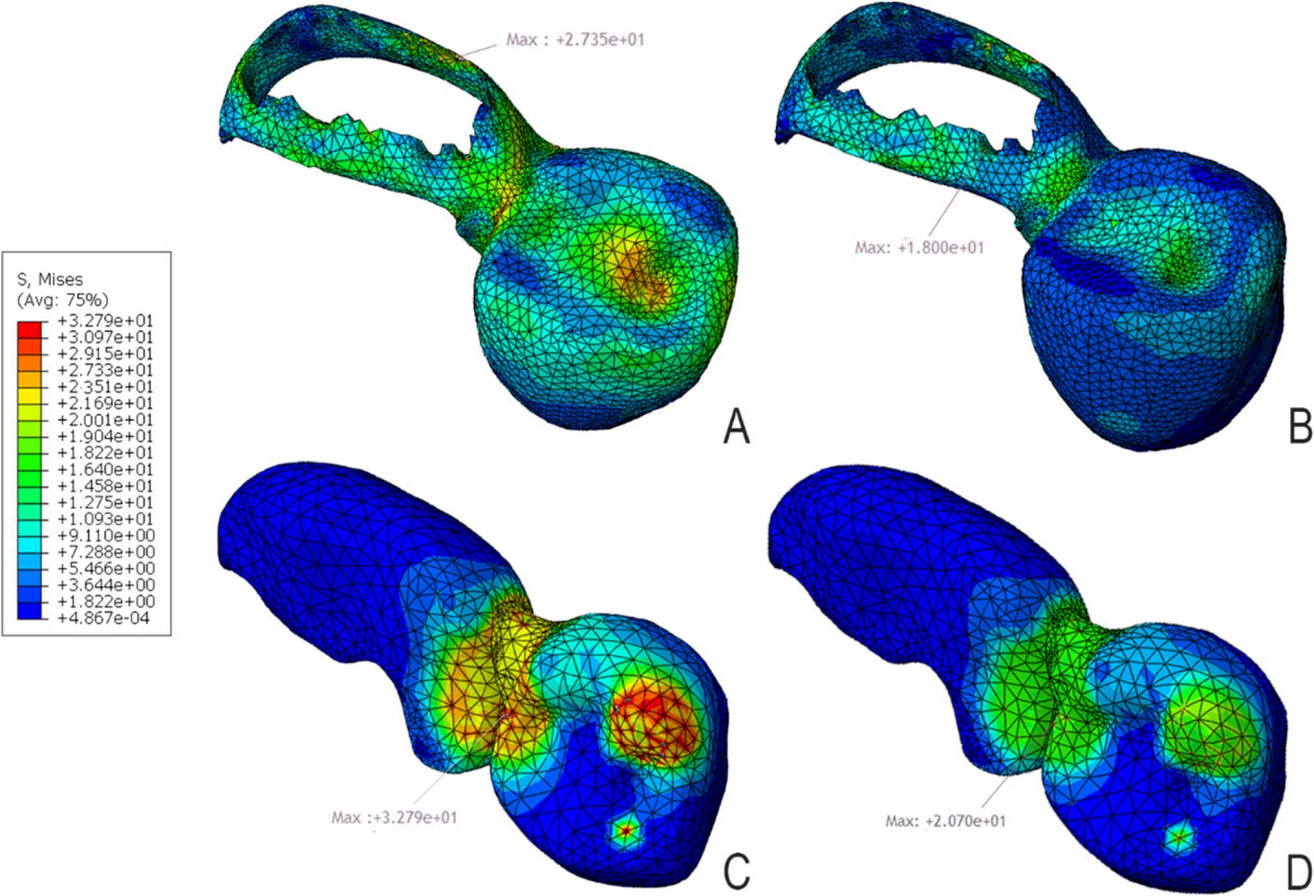
mvM stress distribution pattern for **A** model D1Z, **B** model D1F, **C** model D2Z, and **D** model D2F

For mvM analysis, zirconia showed higher maximum stress values (27.35 MPa for D1 and 32.70 for D2) than FRC (18.00 MPa for D1 and 20.70 for D2). All stress values did not exceed the tensile strength of zirconia (445 MPa) and FRC (380 MPa). The colorimetric maps showed that maximum stresses for model D1 were located at the lingual and inlay wings, while in D2 models were located at the connector area. FRC models showed a more favorable stress distribution than zirconia models. The pontic area showed lower maximum stress values in D1 models than in D2 models.

### Distribution of stresses in luting resin (Fig. 7)

**Fig. 7:**
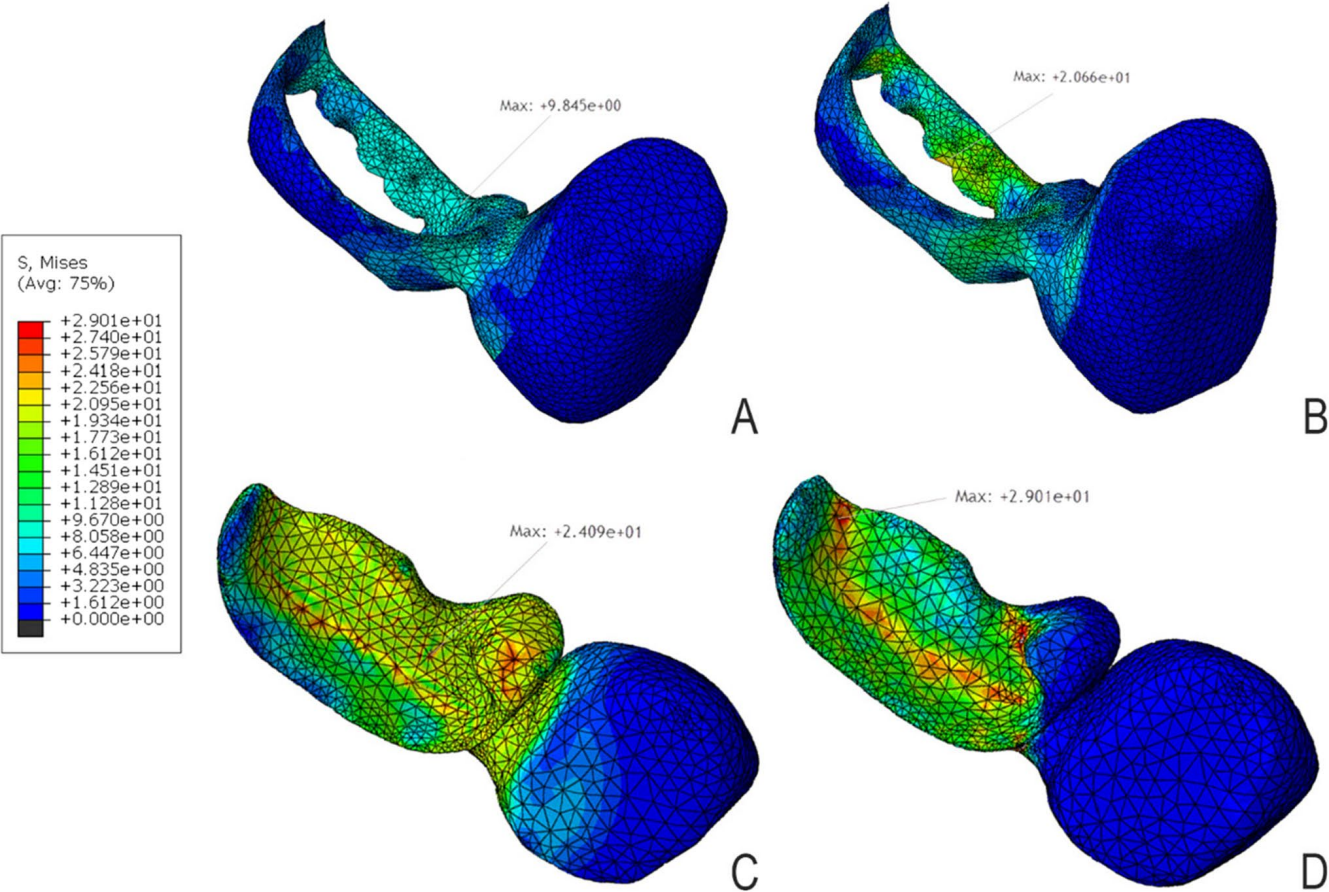
mvM stress distribution pattern for the cement line for **A** model D1Z, **B** model D1F, **C** model D2Z, and **D** model D2F

The luting resin in model D1F showed a significantly higher maximum stress value (20.66 MPa) as compared to model D1Z, which had 9.84 MPa. Both stress values did not exceed the individual cement tensile strength of 30 MPa. For D2 models, the cement in model D2F showed a higher maximum stress value of 29.01 MPa as compared to model D2Z, which had 24.09 MPa; both are near the tensile strength of the resin cement (30 MPa). This means that FRC models showed higher maximum stress values than zirconia models and also revealed that D2 models transmitted higher stresses to the luting resin than D1 models.

## Discussion

The results of this study showed that group D2Z had the highest failure load under static and dynamic loading. Regarding preparation design, the change of material has a significant effect on the failure load. The preparation design did not significantly affect the fracture load when comparing the same material. Therefore, the first hypothesis that the preparation design influences the fracture strength of RBFDPs was rejected. Also, the second hypothesis that the material had no impact on the fracture strength of the restorations was rejected.

Multiple factors influence the fracture strength of prostheses, including the force of applied load, cement type and technique, and elastic modulus of supporting structure. Using a supporting material with a high elastic modulus can increase fracture resistance [42]. The fracture strength of a dental restoration could be more accurate if natural teeth were used as a supporting model [32, 39]. In this study, natural teeth were used, and all specimens were exposed to the same condition of loading and cementation technique. It was a challenge to perfectly standardize the preparations while utilizing natural teeth due to slight variances in the shape and form of the natural teeth. To overcome this limitation, one operator utilized a calibrated surveyor associated with a periodontal probe and a putty index to verify preparation parameters. Finally, the software CAD was employed to ensure that the minimum restoration thickness maintained similarity between the specimen [47].

The choice of the material used for restorations is considered a key factor to enhance the performance of RBFDP. Zirconia, as a control group, was chosen in this study since it is one of the most often used materials for RBFDPs with long-term clinical effectiveness [23]. Zirconia has high tensile strength that enables it to withstand high stress before fracture [48], but increasing the modulus of elasticity of the material can transmit higher stresses to the tooth structure, causing crown and/or root fracture [39]. This can explain the 50% of catastrophic failure found in the specimens from group D1Z.

Different materials with a lower modulus of elasticity are available for CAD/CAM technique [32]. In this study, FRC can promote lower stress magnitude and higher absorption when the load is applied and hence improve the resistance [15]. This could explain the lower values of maximum stresses in tooth structure for FRC subgroups than that measured with zirconia subgroups. In a systematic review [49] that assessed the longevity of FRC FPDs through contemporary clinical evidence, they displayed a high survival rate with predictable performance outcomes. On the other hand, the performance in the long term is unknown. They concluded that FRC FPDs are an effective medium-term option for patients who need to replace a single missing tooth. The present study complements this finding, suggesting that not only the material should be properly selected as well as the prosthesis design.

In vitro simulation of the oral environment with FEA is one of the most reliable methods used in biomechanical investigations of teeth and restorations [18, 32]. For this study, FEA was used to analyze the pattern of stress distribution in translucent zirconia and fiber-reinforced composite cantilever RBFDPs with two different retainer designs. The results can be presented as compressive, shear, tensile, or von Mises stresses transmitted to the investigated structures. Von Mises stresses are a mix of all these stresses that are affected by the applied force. In complex structures with multiple materials, this criterion can be an appropriate indicator of failure since they can show signs of potential damage [31, 33].

The lingual cups (nonfunctional) were chosen to be covered so as to decrease the load applied to the restorations. According to the literature, the intraoral bite forces are in the range of 216–847 N [9, 50]. The mean unilateral bite force in the premolar area is 70% lower than the molar region, with an average of 210–420 N [42]. In another study [51], the mean value of the maximum bite force is around 353 N in men, while in women it is 218 N. In one study [50] discussing the effect of clenching intensity on the biting force in the premolar region, the results showed values from 450 to 660 N. According to our results, these designs cannot be used in case of clenching and bruxism. Occlusal forces rarely reach 45 N during normal chewing, equivalent to the mean of molar bite force [9]. In one study [52], zirconia FDPs were loaded with 50 N in a dynamic fatigue test. Based on these investigations, the specimens evaluated in this study were dynamically loaded at 50 N. To replicate the physiological aging of dental cement clinically, thermo-cycling was conducted. This method can accelerate the aging of dental materials and appears to be valid for in vitro tests. Most resin cement showed a considerable loss in mechanical properties following thermo-cycling [40].

The results of a previous in vitro study [15] evaluated the effect of the retainer design on the fracture resistance of cantilever glass FRC RBFDP. The authors reported higher fracture values than our result. This can be justified because the specimens from the previous study were not artificially aged (mechanical loading and thermo-cycling) as in the present study. The fracture resistance test after artificial aging more accurately resembles the clinical reality than without artificial aging for long-term evaluation. Regarding the inlay ring retainer (D1), the proximal and inlay boxes are within the dentin, therefore, a reduced adhesive area in contact with enamel was present compared to the lingual coverage retainer (D2). Despite that these boxes can increase the retention and resistance form.

The FEA showed that D1 models transmitted more stresses to the tooth structure than the D2 models concentrated in CEJ and in the boxes; this can explain the catastrophic failure in the ring inlay design. The FEA also showed higher stress magnitude in the cement layer with FRC, so debonding occurred; however, zirconia transmitted less stress to the cement line. Regarding the lingual coverage retainer (D2), no feature of retention or resistance was performed, and the retention was dependent only on the adhesion with enamel, so this design showed more adhesive failures than D1. In contrast, this design transmitted less stress to the tooth structure, and no catastrophic failure was recorded in both subgroup. For zirconia subgroups, a significantly higher failure load was recorded in comparison with FRC subgroups, with more stresses transmitted to the tooth structure. While in FRC subgroups, higher stresses were transmitted to the cement, corroborating the higher in vitro debonding rate.

However, the selection of the case and preparation designs are essential factors in the effectiveness of this type of restoration. One of the limitations of the present study was that the load was applied vertically only without resembling the lateral forces as in the oral cavity. The restorations were subjected to the artificial thermo-mechanical aging protocol that only simulates 1 year of clinical services. Other studies with increased thermo-mechanical aging are required. Also, FRC was used as full restoration and not covered by veneering material, the effect of veneering material was not discussed in this study. The finite element method was performed with isotropic materials without defect incorporation and with ideal interfaces. Therefore, it is important to be aware of the fact that the reported values cannot be considered absolute values, and the main purpose was to compare the biomechanical behavior of different designs, interpreting the results from both FEA and mechanical testing simultaneously to reduce the limitations of a single method [45, 46]. Additionally, despite all the limitations, the results are still comparable and can be useful for further study development.

## Conclusions

Under the limitations of the study, the following conclusions were drawn:

1. The lingual coverage retainer (D2) enhanced the biomechanical performance within the restoration-tooth complex when used to replace a missing mandibular premolar compared to the inlay ring retainer (D1);
2. Both zirconia and FRC can be used as cantilever RBFDP in the premolar area. Considering the failure mode and tooth stress, FRC is a promising treatment option when manufacturing a cantilever RPFDP;
3. The higher the modulus of elasticity, the greater the stresses of the m RBFDP. However, in all examined models, the materials’ tensile strength was not exceeded during loading.

## Data Availability

The datasets used and/or analyzed during the current study are available from the corresponding author upon reasonable request.

## Abbreviations

RBFDPs: Resin bonded fixed dental prosthesis
FRC: Fiber reinforced composite
Y-TZP: Yttria-stabilized tetragonal zirconia polycrystal
FEA: Finite-element analysis
CAD/CAM: Computer-aided design/computer-aided manufacturing
SD: Standard deviation
UTM: Universal testing machine
